# Treatment Efficacy and Compliance in Patients with Diabetic Macular Edema Treated with Ranibizumab in a Real-Life Setting

**DOI:** 10.1155/2018/4610129

**Published:** 2018-04-18

**Authors:** Anne-Laurence Best, Franck Fajnkuchen, Sylvia Nghiem-Buffet, Typhaine Grenet, Gabriel Quentel, Corinne Delahaye-Mazza, Salomon Y. Cohen, Audrey Giocanti-Aurégan

**Affiliations:** ^1^Ophthalmology Department, Avicenne Hospital, DHU Vision and Handicaps, APHP, Paris 13 University, Bobigny, France; ^2^Centre d'Imagerie et de Laser, Paris, France; ^3^Ophthalmology Department, Centre Hospitalier Intercommunal de Créteil, Paris Est University, Créteil, France

## Abstract

**Purpose:**

To assess real-life efficacy of ranibizumab and treatment compliance of patients with vision loss secondary to diabetic macular edema (DME).

**Methods:**

A retrospective study was conducted in DME patients treated with ranibizumab. Patients were monitored every 4 weeks for visual acuity (VA) and central retinal thickness (CRT) by SD-OCT. All patients received a loading dose of 3 monthly injections followed by retreatments on an as-needed basis. The primary endpoint was the change in VA at M12. Patient compliance to the follow-up and the correlation between the injection number and VA were also investigated. Compliance was compared to that of neovascular age-related macular degeneration (nAMD) patients.

**Results:**

Seventy-two eyes of 55 consecutive DME patients were included. At baseline, the mean VA was 56.5 letters and CRT was 470 *μ*m. At M12, the mean VA was 63.4 letters (*p* < 0.0001), 31.1% of patients had a VA > 70 letters, the mean VA change was +6.9 letters, and the mean CRT was 361.9 *μ*m (*p* = 0.0001) after a mean number of 5.33 intravitreal injections. In patients who received ≥7 injections, the VA gain and final VA were significantly higher than in patients who received <7 injections. At M12, 25.45% of DME patients were lost to follow-up versus 16.8% of nAMD patients (*n* = 55).

**Discussion/Conclusion:**

Our study confirms the real-life efficacy of ranibizumab in DME at M12 and the need for a large number of injections to achieve better visual outcomes. We also showed a trend to a lower compliance in diabetic versus nAMD patients.

## 1. Introduction

Diabetic macular edema (DME) is the leading cause of decreased vision in diabetic patients with a prevalence of 4.8% [1]. Its management has improved over the last ten years with the increased availability of therapeutic agents. Laser photocoagulation has long been the reference treatment and has led to a 50% reduction in visual acuity (VA) decrease at 3 years, but this improvement is not sustained over the long term [2]. Thereafter, intravitreal injections (IVI) of corticosteroids have shown promising results [3–6] but their side effects limit their benefits [7, 8]. Ranibizumab was the first anti-VEGF agent to show a benefit in terms of VA in the treatment of central DME [9–12] in Phase III studies. In these pivotal studies, the VA gain over the first year varies from +6.8 to +12 letters with a number of IVI ranging between 7 and 12. The visual gain and IVI number depend on the treatment regimen and follow-up strategies used.

The aim of this study was to assess the efficacy and safety of ranibizumab for the treatment of DME in a real-life setting in a French private practice.

## 2. Methods

All consecutive patients with vision loss secondary to DME who received their first IVI of ranibizumab 0.5 mg between June 2012 and June 2015 in a private ophthalmology center specialized in retina diseases, CIL (Center for Imaging and Laser) in Paris, were retrospectively included. This study was conducted in accordance with the tenets of the Declaration of Helsinki, and an informed consent was obtained from patients. Approval was obtained from the France Macula Federation ethics committee.

Inclusion criteria were patients ≥ 18 years old, with type 1 or 2 diabetes with vision loss due to center-involved DME. Both eyes of the same patient could be included.

Exclusion criteria were history of another vitreous or retinal pathology, presence of macular ischemia, stroke or cardiac failure ≤ 3 months before inclusion, and ocular surgery ≤ 6 months before inclusion.

For each patient, the systemic data were collected (diabetes type and duration, HbA1C, blood pressure, dyslipidemia, presence of nephropathy, macroangiopathy, sleep apnea syndrome, and type of treatment).

At baseline and during the follow-up, all patients underwent a complete ophthalmologic examination with best-corrected visual acuity (BCVA) measurement according to the ETDRS scale and slit-lamp and noncontact fundus examination (SuperField Volk). Angiography (Spectralis, Heidelberg Engineering, Heidelberg, Germany) was performed to rule out macular ischemia and to assess the stage of diabetic retinopathy (DR). Spectral-domain optical coherence tomography (SD-OCT) (Spectralis, Heidelberg Engineering, Heidelberg, Germany) was performed to measure the central macular thickness (CRT) and macular volume (MV) during the follow-up. DME was defined by a CRT ≥ 300 *μ*m.

The treatment regimen followed the 2012 European guidelines for ranibizumab use modified in 2014 [13, 14]. Patients received 3 monthly IVI of ranibizumab during the loading phase, followed by reinjection according to a pro re nata (PRN) regimen. Patients were monitored every 4 weeks with BCVA measurement, fundus examination, and CRT measurement. A decrease in BCVA > 5 letters and/or a CRT > 300 *μ*m were indications for retreatment. In the absence of BCVA improvement after the loading phase, treatment was discontinued. Patients with a VA gain < 5 letters or a CRT improvement < 10% from baseline values after 3 IVI were considered as nonresponders.

The primary endpoint was the change in BCVA between baseline and month 12 of follow-up (M12).

Secondary endpoints were the CRT, MV after the loading phase and at M12, number of IVI in the first year of follow-up, and the assessment of patient compliance. Compliance was assessed through 2 parameters: the prevalence of patients lost to follow-up, that is, patients who stopped their follow-up before the end of the first year, and the prevalence of patients with an irregular follow-up, that is, patients who did not attend the required appointments and missed their examination between M12 and M14, but continued their treatment. The compliance of DME patients was compared to that of a series of consecutive neovascular age-related macular degeneration (nAMD) patients treated with ranibizumab for one year in the same center, during the same period.

### 2.1. Statistical Analysis

A matched Student parametric test was used for statistical analysis, and a *p* value < 0.05 was considered significant. For prevalence comparison, a Fisher's exact test was performed. The statistical analysis was carried out using Prism 7 software.

## 3. Results

Seventy-two eyes of 55 patients treated with ranibizumab injections were included. Seventeen patients (30.9%) had bilateral DME, and 38 patients (69.1%) had unilateral DME. The mean DME duration before the first injection was 20.2 months.

The mean follow-up duration after the first IVI was 19.6 months (±11.39 months), with a median of 17.87 months. Baseline patient characteristics are presented in Table 1.

Diabetic retinopathy (DR) was mild nonproliferative DR (NPDR) in 5 eyes (7%), moderate NPDR in 18 eyes (25%), severe NPDR in 20 eyes (27.8%), and proliferative DR in 8 eyes (11.1%). Laser photocoagulation had been previously performed in 21 eyes (29.1%).

Twenty-seven eyes (37.5%) were not treatment naive: 26 eyes had received macular laser therapy and 1 eye had been treated with IVI of triamcinolone in 2004 prior to inclusion. Forty-five eyes (62.5%) were treatment naive (Table 2).

### 3.1. Functional Outcomes

**Figure 1:**
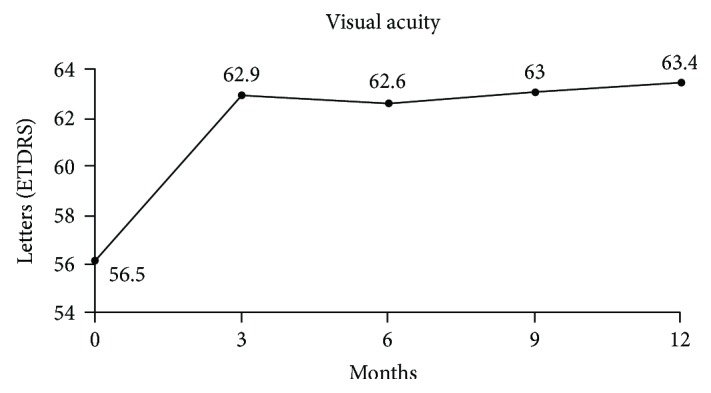
Mean change in best-corrected visual acuity over the first year of follow-up.

The mean baseline BCVA was 56.5 ± 11.9 ETDRS (±SD) letters. Five out of the 72 (6.9%) eyes had a baseline BCVA score > 70 ETDRS (Table 3, Figure 1).

The mean BCVA gain was +6.4 ± 7.3 letters at M3 (*p* < 0.0001), +6.1 ± 16.7 letters at M6 (*p* < 0.0001), +6.5 ± 8.5 letters at M9 (*p* < 0.0001), and +6.9 ± 10.2 ETDRS letters at M12 (*p* < 0.0001). After one year of treatment, 37.8% (17/45) of patients had a VA gain ≥ 10 letters and 22.2% (10/45) had ≥15 letters and 31.1% (14/45) had reached the BCVA threshold of >70 letters versus only 6.9% at baseline.

At the end of the first year of follow-up, 2 eyes had lost ≥10 letters.

### 3.2. Anatomical Outcomes

**Figure 2:**
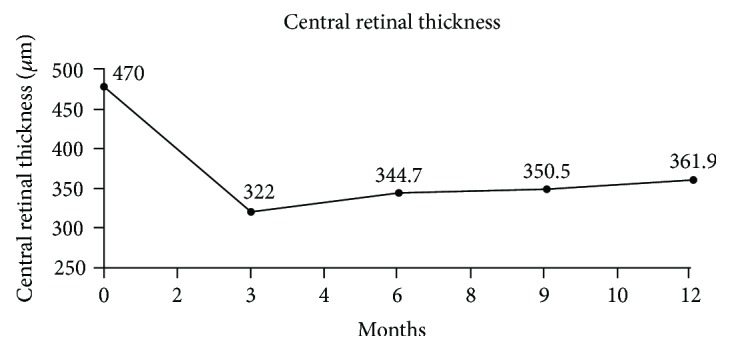
Mean change in central retinal thickness over the first year of follow-up.

The mean baseline CRT was 470 *μ*m (±134.5). The mean CRT change was −148 *μ*m (±177) at M3 and −108.1 *μ*m (±176) at M12 (Table 3, Figure 2). CRT was <300 *μ*m in 40% (18/45) of eyes at M12.

**Figure 3:**
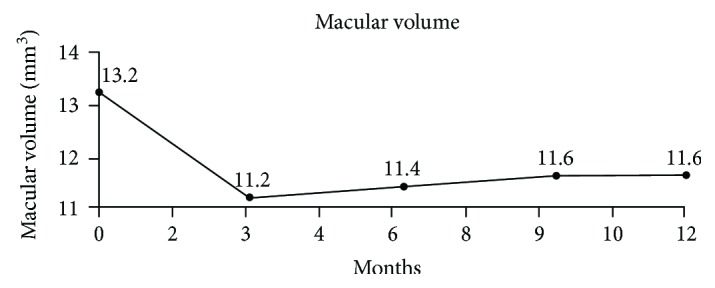
Mean change in macular volume over the first year of follow-up.

The baseline MV was 13.2 mm^3^. The mean change in MV was −2 ± 1.6 mm^3^ at M3 and −1.6 ± 1.6 mm^3^ at M12 (Table 3, Figure 3).

### 3.3. Number of Intravitreal Injections

55 patients (72 eyes) received a mean number of 5.33 ± 2.1 injections of ranibizumab over the first year. Nineteen eyes had a follow-up of two years with a mean number of 10.84 IVI.

### 3.4. Compliance with Treatment

Nine (16.4%) and 14 (25.45%) patients (10 and 16 eyes) were lost to follow-up at M6 and M12, respectively. As a result, 41 patients (56 eyes) had at least 12 months of follow-up, but only 33 out of the 55 patients (60%, 45 eyes) attended the control consultation scheduled between the 12th and 14th month, the others were seen later (i.e., 8 patients—14.5%—had an irregular follow-up).

### 3.5. Baseline Characteristics and Compliance of nAMD Patients

Fifty-five consecutive patients with nAMD seen in the same private practice and requiring ranibizumab IVI since January 2013 and followed over 12 months were also included. We included 41 women and 14 men with a mean age of 85.3 (±6.3). The mean baseline visual acuity was 61.6 (±13.5) letters.

A mean number of 7.38 consultations were carried out over one year. A mean number of 4.5 IVI were administered over the first year. Only 16.8% of patients were lost to follow-up at one year.

### 3.6. Subgroup Analysis

#### 3.6.1. Subanalysis according to the Number of IVI at 1 Year

Two subgroups of patients were defined based on the number of IVI administered during the first year: one group received <7 IVI (*n* = 30 eyes) and one received ≥7 IVI (*n* = 15 eyes). Patients who received <7 IVT had a baseline BCVA of 55.5 letters and a visual gain of +5.43 letters versus a baseline BCVA of 57.1 letters (*p* = 0.09) and a visual gain of +11.19 letters for patients who received ≥7 IVT. At one year, a mean BCVA of 60.96 ± 15.66 letters was achieved in the group that received <7 IVT versus 68.26 ± 6.99 letters in the group with ≥7 IVT (*p* = 0.04).

#### 3.6.2. Functional Response Subanalysis at 1 Year

Two subgroups were defined according to the functional response after one year of treatment. A subgroup of good responders (*n* = 8 eyes) was defined as a BCVA gain > 15 letters at 1 year, and a subgroup of poorer responders was defined by a BCVA gain ≤ 15 letters. The group of poorer responders received fewer IVI than the group of good responders (mean IVI number: 5.59 versus 6.5) over the first year (*p* = 0.03). In the good responder group, the baseline BCVA was 46.9 letters and 58.2 letters in the poorer responders (*p* = 0.047).

### 3.7. Safety

No case of endophthalmitis was reported during the follow-up. One patient with type 2 diabetes had a stroke 6 weeks after the last IVI. This patient subsequently underwent a complete ophthalmologic evaluation, and the decision was made to discontinue IVI.

## 4. Discussion

The results of our study confirm the efficacy of ranibizumab for the treatment of DME responsible for vision loss in a real-life setting with a VA gain of +6.9 ± 10.2 letters after a mean number of 5.33 IVT over the first year of follow-up.

However, our functional results at 1 year are slightly lower than those reported in pivotal [10] and http://drcr.net studies [8, 12, 15] which show a gain from +6.5 to +12 letters at M12. This discrepancy could probably be due to an insufficient number of injections in our real-life series. Indeed, in our study, patients received 5.33 IVI with a mean annual number of 7.68 consultations, compared to 7–9.4 IVI in pivotal and DRCR.net studies with a number of consultations generally higher than that of our patients.

In the RISE and RIDE studies [11], patients were injected monthly for 36 months. In this case, the VA gains ranged from +11.9 to +12 letters [16] after one year of follow-up. In Europe, in the RESTORE study [10], with a strict monthly monitoring, the visual gain was +6.8 letters at the end of the first year of treatment with a mean number of 7 IVI. Patients were treated according to a PRN regimen, and the retreatment criterion was strictly functional.

In the DRCR.net studies [8, 12, 15], ranibizumab IVI were administrated according to a PRN regimen with retreatment based on functional and anatomical outcomes with severe retreatment criteria during the first 6 months to achieve a VA of 20/20 or a dry retina. Thus, patients usually received 5 or 6 injections during the first 24 weeks. With this type of treatment and monitoring every 4 weeks, a gain of +9 letters after 9 IVI was observed with protocol I and +11.2 letters after 10 IVI with protocol T. However, in our study, despite consultations scheduled every 4 weeks, the time between each consultation was longer than 4 weeks in patients who completed the one-year follow-up since they only attended a mean number of 7.68 visits over 12 months.

A clear difference in terms of visual outcomes between the real-life setting and pivotal studies has already been observed in nAMD patients treated with ranibizumab. In nAMD, the MARINA [17] and ANCHOR [18] pivotal studies have shown VA gains ranging between +7.2 and +11.3 letters at one year. The PrONTO study [19] has shown a sustained VA improvement with a personalized PRN regimen and retreatment based on functional and anatomical outcomes allowing a gain of +9.3 letters at one year with twice fewer injections but with a proper monthly follow-up. Real-life studies have shown a smaller improvement with a gain of +4.4 letters at one year for the LUMINOUS [20] study. Another real-life study conducted in our center has shown an even lower visual gain of +0.7 letter after 3.79 IVI and 8.06 consultations over the first year under a PRN regimen, and the authors have concluded on the need for a more regular follow-up with a strict 4-week interval between each consultation. These real-life studies have stressed that there could be a difference in terms of functional outcomes between data from randomized studies with a strict monitoring and treatment protocols and the real-life conditions.

In DME, differences in functional outcomes seem less significant than in nAMD between pivotal and real-life results. The ADMOR real-life study [21] has investigated the efficacy of ranibizumab in patients with DME in South Asia. The results showed a gain of +8.5 letters at 1 year with a mean number of 7 ± 2 IVI over the first year. In this study, patients were not strictly monitored every 4 weeks and attended a mean number of 10 ± 2 visits during their follow-up. Patients in the ADMOR study had a more severe DME, with an initial VA less than ours (55.3 ± 13.4 letters), and a higher baseline CRT (532 ± 129 *μ*m). Another real-life study by Hrarat et al. [22] has reported a gain of +10.7 ± 16.9 letters after 12 months of treatment with a mean number of 5.4 ± 1.9 IVI and 8.8 ± 2.5 visits during the follow-up. The mean baseline VA was 48.3 ± 17 letters, and the baseline CRT was 519.7 ± 157.3 *μ*m. This very low baseline VA could explain their high VA gain [16]. A Swedish real-life study by Granström et al. [23] assessing the efficacy of a 12-month treatment with ranibizumab in DME, retrospectively conducted in two ophthalmic departments using a PRN regimen, has reported a gain of +5.2 letters after 12 months of treatment, but the mean number of injections was not specified. Patients had an initial VA greater than ours (65.0 ± 12.1 letters) with a lower initial CRT: 403 ± 122 *μ*m.

In our study, with a stricter follow-up and treatment regimen, the VA gains could have probably been greater. This finding is reinforced by a statistically significant correlation between the VA gain and the number of IVI in our study. Patients with more than 7 IVI had a higher VA gain than those who received less than 7 IVI (*p* < 0.04). In addition, the number of injections was greater in the group of patients who had a gain greater than 15 letters compared to the group that did not exceed this threshold (*p* < 0.03).

These results encourage us to adopt a strict follow-up and highlight the need for a regular follow-up by providing appropriate information to patients. Appropriate information is indeed important as the compliance of diabetic patients may be low. Thus, in our series, it should be noted that a significant number of patients were lost to follow-up (25.45% of patients), suggesting that some diabetic patients are poorly compliant. The small percentage of patients (60%) who attended the 12-month consultation supports this hypothesis. This discrepancy between real-life and pivotal studies stresses that real-life studies are necessary to assess the true efficacy of a treatment and to understand the factors limiting efficacy.

The treatment regimen of DME represents a real burden for patients and their family, and diabetic patients must also attend different medical consultations with several specialists and this may be a barrier to a monthly follow-up. Thus, this burden of consultations not only with ophthalmologists could contribute to the lower compliance of diabetic patients compared to that of nAMD patients. Indeed, we assessed in the same private practice 55 consecutive patients with nAMD requiring ranibizumab IVI and followed them over 12 months. They attended a mean number of 7.38 consultations and received a mean number of 4.5 IVI over the first year. Only 16.8% of patients were lost to follow-up at one year versus 25.45% in our series of diabetic patients (*p* = 0.6).

Different assumptions may be made regarding the lower compliance of diabetic patients compared to AMD patients: the fact that (i) DME is part of a chronic extraophthalmological disease, diabetes, which, because of its chronicity, may lead to a lassitude with regard to the disease; (ii) the loss of vision is progressive in DME compared to the sudden and often deeper vision loss in nAMD; (iii) diabetic patients are younger and often in the working age, making them less available than nAMD patients who are often retired; and (iv) the cost of the treatment, which may also be a barrier, in particular in a private center where patients must advance the cost. Other studies are needed to confirm the lower compliance of DME patients compared to nAMD patients.

Based on our findings and the results of the literature [24], it seems essential to adopt the treatment regimen to specificities of the diabetic population and to patient availability and preferences after information and, in the case of patients who cannot follow a strict monthly regimen to choose the appropriate treatment, for instance, a treat-and-extend regimen, providing the same visual outcomes with a lower number of consultations [24] and thus, even despite a possible overtreatment for a few patients.

In conclusion, our real-life study shows a VA improvement in patients with DME, with however a slightly lower gain than that found in pivotal studies after a lower number of IVI. This discrepancy between results obtained in a real-life setting and pivotal studies is not as important as in nAMD despite a higher compliance of nAMD patients in a real-life setting.

This study also shows that the visual outcomes correlate with the number of IVI, and that a strict monthly follow-up is challenging in the real life.

## Figures and Tables

**Table 1:** Baseline characteristics of patients.

Patient number	*n* = 55
Sex	
Men	*n* = 34 (61.8%)
Women	*n* = 21 (38.2%)
Type of diabetes	
Type 1	*n* = 8 (15.5%)
Type 2	*n* = 47 (85.5%)
Age (years), mean (±SD^∗^)	66.7 (±9.59)
Duration of diabetes (years), mean (±SD)	18.1 (±13.29)
HbA1c, mean (±SD)	7.4% (±1.25)
Insulinotherapy	*n* = 20 (36%)
High blood pressure	*n* = 34 (61.8%)
Dyslipidemia	*n* = 14 (25%)
Nephropathy	*n* = 15 (27%)
Macroangiopathy	*n* = 2 (3.6%)
Sleep apnea syndrome	*n* = 2 (3.6%)

^∗^SD: standard deviation.

**Table 2:** Baseline features of retinopathy, maculopathy, and ophthalmologic history.

Eye number	*n* = 72
NPDR	
Mild	5 (7%)
Moderate	18 (25%)
Severe	20 (27.7%)
PDR	8 (11.1%)
Laser photocoagulation	
PRP	
Ongoing	22 (30.5%)
Completed	21 (29.1%)
Focal/grid	26 (36.1%)
Intravitreal injection history	
Corticosteroids	1 (1.3%)
DME duration (months): mean (±SD)	20.2 (±25.13)
Pseudophakic	18 (25%)
Vitreomacular surgery	4 (5.6%)
Epiretinal membrane	10 (13.8%)
High intraocular pressure history	4 (5.5%)

NPRD: nonproliferative diabetic retinopathy; PDR: proliferative diabetic retinopathy; PRP: panretinal photocoagulation; SD: standard deviation; *n*: number of eyes.

**Table 3:** Best-corrected visual acuity (BCVA), central retinal thickness (CRT), and macular volume (MV) over the first year of follow-up.

	Baseline	Month 3	Month 6	Month 9	Month 12
Number of eyes	*n* = 72	*n* = 60	*n* = 58	*n* = 52	*n* = 45
BCVA (ETDRS letters ± SD)	56.5 ± 11.9	62.9 ± 12.4	62.6 ± 13.0	63.0 ± 12.2	63.4 ± 13.8
CRT (*μ*m ± SD)	470 ± 134.5	322 ± 97.8	344.7 ± 122.8	350.5 ± 99	361.9 ± 124.8
MV (mm^3^ ± SD)	13.2 ± 2.4	11.2 ± 1.5	11.4 ± 1.7	11.6 ± 1.7	11.6 ± 1.6
BCVA > 70 letters	5 (6.9%)	22 (36.6%)	18 (31%)	16 (30.7%)	14 (31.1%)
		0–3 months	0–6 months	0–9 months	0–12 months
Number of eyes	*n* = 72	*n* = 60	*n* = 58	*n* = 52	*n* = 45
BCVA gain (ETDRS letters ± SD)		+6.4 ± 7.3^∗^	+6.1 ± 16.7^∗^	+6.5 ± 8.5^∗^	+6.9 ± 10.2^∗^
Change in CRT (*μ*m ± SD)		−148 ± 177	−125.3 ± 177	−119.5 ± 143	−108.1 ± 176
Change in MV (mm^3^ ± SD)		−2 ± 1.6	−1.8 ± 1.8	−1.7 ± 1.4	−1.6 ± 1.6
Gain ≥ 10 letters		22 (30.5%)	19 (26.3%)	18 (25%)	17 (37.8%)
Gain ≥ 15 letters		9 (12.5%)	11 (15.2%)	5 (6.9%)	10 (22.2%)
Loss ≥ 10 letters		1 (1.3%)	6 (8.3%)	2 (2.7%)	3 (4.1%)
Loss ≥ 15 letters		0	3 (4.1%)	0	2 (2.7%)

^∗^ *p* < 0.0001.
